# Supplementary material for: Drought and intimate partner violence towards women in 19 countries in sub-Saharan Africa during 2011-2018: A population-based study
Source: PLoS Med. 2020 Mar 19;17(3):e1003064. doi: 10.1371/journal.pmed.1003064 (PMC7081984; doi:10.1371/journal.pmed.1003064)
Supplement: S1 Table — (DOCX) [file pmed.1003064.s002.docx]

**S1 Table. List of surveys included in analysis**

| **Country** | **Survey Phase** | **Year** | **Number in analytic sample** |
| --- | --- | --- | --- |
| Sierra Leone | DHS-VI | 2013 | 3,877 |
| Togo | DHS-VII | 2013-14 | 4,763 |
| Benin | DHS-VII | 2017-2018 | 3,856 |
| Cote d’Ivoire | DHS-VI | 2011-12 | 4,151 |
| Cameroon | DHS-VI | 2011 | 3,396 |
| Gabon | DHS-VI | 2012 | 3,112 |
| Chad | DHS-VII | 2014-15 | 3,174 |
| Democratic Republic of Congo | DHS-VI | 2013-14 | 4,557 |
| Rwanda | DHS-VII | 2014-15 | 1,622 |
| Burundi | DHS-VII | 2016-17 | 6,364 |
| Uganda | DHS-VII | 2016 | 6,119 |
| Kenya | DHS-VI | 2014 | 3,775 |
| Tanzania | DHS-VII | 2015-16 | 6,452 |
| Malawi | DHS-VII | 2012-13 | 4,537 |
| Mozambique | DHS-VI | 2011 | 4,617 |
| Zimbabwe | DHS-VII | 2015 | 4,845 |
| Zambia | DHS-VI | 2013-14 | 7,577 |
| Namibia | DHS-VI | 2013 | 1,192 |
| Angola | DHS-VII | 2015-16 | 6,004 |
