# Supplementary material for: Drought and intimate partner violence towards women in 19 countries in sub-Saharan Africa during 2011-2018: A population-based study
Source: PLoS Med. 2020 Mar 19;17(3):e1003064. doi: 10.1371/journal.pmed.1003064 (PMC7081984; doi:10.1371/journal.pmed.1003064)
Supplement: S2 Table — (DOCX) [file pmed.1003064.s003.docx]

| **S2 Table. Definition and dimensions of the outcomes considered in this analysis.** | | |
| --- | --- | --- |
| **Outcome** | **Definition** | **Dimensions*** |
| Respondent self-reported any control issue | Binary indicator representing endorsement of any control issue dimension | - Husband/partner is jealous if respondent talks to other men - Husband/partner accuses respondent of unfaithfulness - Husband/partner does not permit respondent to meet female friends - Husband/partner tries to limit respondent’s contact with family - Husband/partner insists on knowing where respondent is - Husband/partner does not trust respondent with money |
| Respondent ever experienced emotional violence in the past 12 months | Binary indicator representing endorsement of any emotional violence dimension in the past 12 months | - Ever been humiliated by husband/partner - Ever been threatened with harm by husband/partner - Ever been insulted or made to feel bad by husband/partner |
| Respondent ever experienced physical violence in the past 12 months | Binary indicator representing endorsement of any physical violence dimension in the past 12 months | - Ever been pushed, slapped or shook or had something thrown by husband/partner - Ever been slapped by husband/partner - Ever been punched with fist or hit by something harmful by husband/partner - Ever been kicked or dragged by husband/partner - Ever been strangled or burnt by husband/partner - Ever been threatened with knife/gun or other weapon by husband/partner - Ever had arm twisted or hair pulled by husband/partner |
| Respondent ever experienced sexual violence in the past 12 months | Binary indicator representing endorsement of any sexual violence dimension in the past 12 months | - Ever been physically forced into unwanted sex by husband/partner - Ever been forced into other unwanted sexual acts by husband/partner - Ever been physically forced to perform sexual acts respondent didn't want to |
| *Surveys also include country-specific dimensions not listed here. | | |
