# Supplementary material for: Drought and intimate partner violence towards women in 19 countries in sub-Saharan Africa during 2011-2018: A population-based study
Source: PLoS Med. 2020 Mar 19;17(3):e1003064. doi: 10.1371/journal.pmed.1003064 (PMC7081984; doi:10.1371/journal.pmed.1003064)
Supplement: S3 Table — (DOCX) [file pmed.1003064.s004.docx]

| **S3 Table. Associations between drought and IPV among women aged 15-49 in pooled analysis (drought considered binary variable) (n = 83,990).** | | |
| --- | --- | --- |
| *Outcome* | Unadjusted | Adjusted |
| At least 1 control issue reported | 1.7* (0.3, 3.0) | 1.5* (0.1, 2.8) |
| Ever experienced emotional violence in previous 12 months | -0.5 (-1.6, 0.6) | -0.6 (-1.7, 0.5) |
| Ever experienced physical violence in previous 12 months | 0.8* (0.2, 1.3) | 0.7* (0.1, 1.3) |
| Ever experienced sexual violence in previous 12 months | 0.7* (0.1, 1.3) | 0.7* (0.1, 1.3) |
| Coefficients are presented as marginal risk difference estimates in percentage points from logistic regression models with 95% confidence intervals in parentheses. The unadjusted model includes country-level fixed effects. The adjusted model includes age category, literacy, marital status, number of births, household size, rural, husband/partner’s age, and husband/partner’s education. Standard errors are clustered at the EA level.  Asterisks denote level of significance **p<0.01 **p<0.05 | | |
