# Supplementary material for: Drought and intimate partner violence towards women in 19 countries in sub-Saharan Africa during 2011-2018: A population-based study
Source: PLoS Med. 2020 Mar 19;17(3):e1003064. doi: 10.1371/journal.pmed.1003064 (PMC7081984; doi:10.1371/journal.pmed.1003064)
Supplement: S4 Table — (DOCX) [file pmed.1003064.s005.docx]

| **S4 Table. Associations between drought and IPV among all women aged 15-49 in pooled analysis (drought as binary variable) (n = 83,990).** | | | | |
| --- | --- | --- | --- | --- |
|  | At least 1 control issue reported | Emotional violence in past 12 months | Physical violence in past 12 months | Sexual violence in past 12 months |
| *Covariate* | Odds ratio (95% CI) | Odds ratio (95% CI) | Odds ratio (95% CI) | Odds ratio (95% CI) |
| Drought | 1.07* (1.01, 1.15) | 0.96 (0.89, 1.04) | 1.15* (1.03, 1.28) | 1.17* (1.02, 1.35) |
| Age |  |  |  |  |
| 15-19 | REF | REF | REF | REF |
| 20-29 | 1.02 (0.95, 1.10) | 1.14** (1.05, 1.25) | 1.07 (0.92, 1.24) | 0.90 (0.77, 1.05) |
| 30-39 | 0.92 (0.85, 1.00) | 1.06 (0.96, 1.18) | 0.91 (0.76, 1.08) | 0.83 (0.69, 1.00) |
| 40-49 | 0.76*** (0.69, 0.84) | 0.96 (0.85, 1.08) | 0.87 (0.71, 1.06) | 0.73** (0.58, 0.91) |
| Literate | 1.12*** (1.08, 1.17) | 0.97 (0.92, 1.01) | 0.93 (0.86, 1.01) | 0.96 (0.88, 1.05) |
| Married | 0.72*** (0.69, 0.76) | 0.87*** (0.83, 0.92) | 0.76*** (0.70, 0.83) | 0.82*** (0.75, 0.90) |
| Number of births |  |  |  |  |
| 0 | REF | REF | REF | REF |
| 1-2 | 1.01 (0.94, 1.08) | 1.35*** (1.23, 1.48) | 1.22* (1.04, 1.43) | 1.27** (1.06, 1.51) |
| 3-4 | 1.02 (0.95, 1.11) | 1.58*** (1.43, 1.75) | 1.50*** (1.26, 1.78) | 1.34** (1.10, 1.62) |
| 5+ | 1.07 (0.98, 1.16) | 1.78*** (1.60, 1.98) | 1.74*** (1.45, 2.10) | 1.50*** (1.22, 1.86) |
| Household size |  |  |  |  |
| 2-3 | REF | REF | REF | REF |
| 4-5 | 0.99 (0.95, 1.04) | 1.06* (1.01, 1.12) | 0.99 (0.90, 1.09) | 1.08 (0.98, 1.20) |
| 6+ | 0.99 (0.94, 1.04) | 1.08* (1.02, 1.14) | 0.98 (0.88, 1.08) | 1.07 (0.95, 1.19) |
| Rural | 0.83*** (0.79, 0.87) | 0.98 (0.93, 1.03) | 0.85*** (0.79, 0.92) | 0.97 (0.89, 1.07) |
| Husband’s education |  |  |  |  |
| No education | REF | REF | REF | REF |
| Primary | 1.18*** (1.13, 1.25) | 1.14*** (1.08, 1.21) | 1.14* (1.03, 1.26) | 1.11 (0.99, 1.24) |
| Secondary | 1.26*** (1.19, 1.33) | 0.99 (0.93, 1.05) | 1.07 (0.96, 1.20) | 0.96 (0.85, 1.09) |
| Higher | 1.03 (0.95, 1.11) | 0.76*** (0.69, 0.83) | 0.68*** (0.57, 0.81) | 0.53*** (0.43, 0.66) |
| Husband’s age |  |  |  |  |
| 15-19 | REF | REF | REF | REF |
| 20-29 | 1.31** (1.08, 1.60) | 1.00 (0.78, 1.29) | 0.80 (0.56, 1.16) | 0.75 (0.50, 1.13) |
| 30-39 | 1.28* (1.05, 1.56) | 0.97 (0.76, 1.25) | 0.76 (0.52, 1.10) | 0.79 (0.53, 1.20) |
| 40-49 | 1.29* (1.05, 1.58) | 0.94 (0.73, 1.22) | 0.66* (0.45, 0.97) | 0.68 (0.45, 1.03) |
| 50+ | 1.24* (1.01, 1.52) | 0.90 (0.70, 1.17) | 0.60* (0.40, 0.88) | 0.58* (0.38, 0.90) |
| Coefficients are presented as odds ratio estimates from logistic regression models with 95% confidence intervals in parentheses. Models include country-level fixed effects. Standard errors are clustered at the EA level.  Asterisks denote level of significance ***p<0.001 **p<0.01 **p<0.05 | | | | |
