# Supplementary material for: Drought and intimate partner violence towards women in 19 countries in sub-Saharan Africa during 2011-2018: A population-based study
Source: PLoS Med. 2020 Mar 19;17(3):e1003064. doi: 10.1371/journal.pmed.1003064 (PMC7081984; doi:10.1371/journal.pmed.1003064)
Supplement: S5 Table — (DOCX) [file pmed.1003064.s006.docx]

| **S5 Table. Associations between drought and number of IPV outcomes endorsed among all women aged 15-49 in pooled analysis.** | | |
| --- | --- | --- |
| *Exposure* | Unadjusted | Adjusted |
|  | Odds ratio (95% CI) | Odds ratio (95% CI) |
| No Drought | REF | REF |
| Moderate/ Mild Drought | 1.04 (1.00, 1.09) | 1.03 (0.99, 1.08) |
| Severe Drought | 1.15*** (1.08, 1.24) | 1.14*** (1.07, 1.23) |
| Coefficients are presented as odds ratios from ordered logistic regression models with 95% confidence intervals in parentheses. The unadjusted model includes country-level fixed effects. The adjusted model includes age category, literacy, marital status, number of births, household size, rural, husband/partner’s age, and husband/partner’s education. Standard errors are clustered at the EA level.  Asterisks denote level of significance ***p<0.001 **p<0.01 *p<0.05 | | |
