# Supplementary material for: Drought and intimate partner violence towards women in 19 countries in sub-Saharan Africa during 2011-2018: A population-based study
Source: PLoS Med. 2020 Mar 19;17(3):e1003064. doi: 10.1371/journal.pmed.1003064 (PMC7081984; doi:10.1371/journal.pmed.1003064)
Supplement: S6 Table — (DOCX) [file pmed.1003064.s007.docx]

| **S6 Table. Associations between drought and IPV among women aged 15-49 in pooled analysis with each country sequentially removed.** | | | | | | | | |
| --- | --- | --- | --- | --- | --- | --- | --- | --- |
|  | *Outcome* | | | | | | | |
|  | At least 1 control issue reported | | Emotional violence in previous 12 months | | Physical violence in previous 12 months | | Sexual violence in previous 12 months | |
| *Exposure* | Unadjusted | Adjusted | Unadjusted | Adjusted | Unadjusted | Adjusted | Unadjusted | Adjusted |
| Angola Removed | | | | | | | | |
| No drought | REF | REF | REF | REF | REF | REF | REF | REF |
| Moderate/ mild drought | 0.4 (-0.8, 1.6) | 0.0 (-1.0, 1.2) | 0.4 (-0.5, 1.3) | 0.4 (-0.6, 1.3) | 0.7** (0.3, 1.2) | 0.7** (0.2, 1.1) | 0.7** (0.2, 1.1) | 0.6** (0.2, 1.1) |
| Severe drought | 2.9*** (1.2, 4.7) | 2.6** (0.9, 4.4) | 0.7 (-0.7, 2.2) | 0.6 (-0.9, 2.0) | 1.0* (0.2, 1.9) | 1.0* (0.2, 1.8) | 1.2** (0.3, 2.2) | 1.2** (0.2, 2.2) |
| Benin Removed | | | | | | | | |
| No drought | REF | REF | REF | REF | REF | REF | REF | REF |
| Moderate/ mild drought | 0.3 (-0.8, 1.5) | 0.2 (-0.1, 1.4) | 0.5 (-0.4, 1.5) | 0.5 (-0.5, 1.4) | 0.7** (0.3, 1.2) | 0.7** (0.2, 1.2) | 0.9*** (0.4, 1.3) | 0.8** (0.4, 1.3) |
| Severe drought | 3.0** (1.3, 4.7) | 3.0*** (1.3, 4.6) | 0.2 (-1.3, 1.6) | 0.0 (-1.4, 1.5) | 0.9* (0.1, 1.6) | 0.9* (0.1, 1.6) | 1.1* (0.2, 1.9) | 1.0* (0.2, 1.8) |
| Burundi Removed | | | | | | | | |
| No drought | REF | REF | REF | REF | REF | REF | REF | REF |
| Moderate/ mild drought | 0.1 (-1.0, 1.4) | -0.1 (-1.3, 1.0) | 0.4 (-0.6, 1.3) | 0.4 (-0.6, 1.3) | 0.7** (0.3, 1.2) | 0.7** (0.1, 1.5) | 0.8*** (0.4, 1.3) | 0.8*** (0.4, 1.3) |
| Severe drought | 2.9*** (1.3, 4.5) | 2.9*** (1.3, 4.5) | 0.6 (-0.8, 1.9) | 0.4 (-0.9, 1.8) | 0.9* (0.2, 1.5) | 0.8* (0.1, 1.5) | 1.3** (0.5, 2.0) | 1.3** (0.5, 2.1) |
| Cameroon Removed | | | | | | | | |
| No drought | REF | REF | REF | REF | REF | REF | REF | REF |
| Moderate/ mild drought | 0.3 (-0.9, 1.5) | 0.0 (-1.1, 1.2) | 0.4 (-0.5, 1.3) | 0.4 (-0.5, 1.3) | 0.7** (0.2, 1.1) | 0.6** (0.2, 1.1) | 0.7** (0.3, 1.2) | 0.7** (0.3, 1.2) |
| Severe drought | 3.0*** (1.4, 4.7) | 3.0*** (1.4, 4.6) | 0.6 (-0.8, 1.9) | 0.4 (-1.0, 1.7) | 0.8* (0.1, 1.5) | 0.8* (0.1, 1.5) | 1.3** (0.5, 2.1) | 1.2** (0.4, 2.1) |
| Chad Removed | | | | | | | | |
| No drought | REF | REF | REF | REF | REF | REF | REF | REF |
| Moderate/ mild drought | 0.0 (-1.2, 1.2) | -0.3 (-1.4, 0.9) | 0.4 (-0.5, 1.3) | 0.2 (-0.7, 1.2) | 0.6** (0.2, 1.1) | 0.6* (0.1, 1.1) | 0.7** (0.2, 1.2) | 0.7** (0.3, 1.1) |
| Severe drought | 1.6* (0.0, 3.3) | 1.7* (0.0, 3.4) | 0.3 (-1.1, 1.8) | 0.1 (-1.2, 1.6) | 0.8* (0.0, 1.5) | 0.7* (0.0, 1.5) | 1.2** (0.3, 2.1) | 1.1** (0.2, 2.0) |
| Cote d’Ivoire Removed | | | | | | | | |
| No drought | REF | REF | REF | REF | REF | REF | REF | REF |
| Moderate/ mild drought | 0.5 (-0.6, 1.7 | 0.2 (-1.0, 1.3) | 0.4 (-0.5, 1.4) | 0.4 (-0.5, 1.3) | 0.8** (0.3, 1.2) | 0.7** (0.2, 1.2) | 0.8** (0.3, 1.2) | 0.8** (0.3, 1.2) |
| Severe drought | 3.1*** (1.5, 4.7) | 3.0*** (1.4, 4.6) | 0.6 (-0.8, 2.0) | 0.4 (-1.0, 1.8) | 0.9* (0.2, 1.7) | 0.9* (0.1, 1.6) | 1.3** (0.5, 2.2) | 1.3** (0.4, 2.2) |
| Democratic Republic of the Congo Removed | | | | | | | | |
| No drought | REF | REF | REF | REF | REF | REF | REF | REF |
| Moderate/ mild drought | 0.3 (-0.9, 1.5) | -0.1 (-1.2, 1.1) | 0.1 (-0.8, 1.1) | 0.1 (-0.8, 1.0) | 0.6* (0.0, 1.0) | 0.5* (0.0, 0.9) | 0.3 (-0.1, 0.7) | 0.3 (-0.1, 0.7) |
| Severe drought | 2.9** (1.2, 4.6) | 2.8** (1.1, 4.5) | 0.4 (-1.0, 1.7) | 0.2 (-1.2, 1.6) | 0.8* (0.0, 1.5) | 0.7* (0.0, 1.4) | 0.9* (0.2, 1.6) | 0.8* (0.1, 1.6) |
| Gabon Removed | | | | | | | | |
| No drought | REF | REF | REF | REF | REF | REF | REF | REF |
| Moderate/ mild drought | 0.4 (-0.8, 1.6) | 0.1 (-1.1, 1.3) | 0.4 (-0.5, 1.3) | 0.5 (-0.4, 1.5) | 0.7** (0.2, 1.2) | 0.7** (0.2, 1.1) | 0.7** (0.3, 1.2) | 0.7** (0.3, 1.2) |
| Severe drought | 3.1*** (1.4, 4.8) | 3.1*** (1.4, 4.7) | 0.5 (-0.9, 1.9) | 0.5 (-0.9, 1.8) | 0.6 (-0.1, 1.3) | 0.6 (-0.1, 1.3) | 1.3** (0.4, 2.1) | 1.2** (0.4, 2.1) |
| Kenya Removed | | | | | | | | |
| No drought | REF | REF | REF | REF | REF | REF | REF | REF |
| Moderate/ mild drought | 0.4 (-0.8, 1.6) | 0.1 (-1.1, 1.3) | 0.2 (-0.7, 1.2) | 0.2 (-0.7, 1.2) | 0.7** (0.2, 1.1) | 0.7** (0.2, 1.1) | 0.8** (0.3, 1.2) | 0.7** (0.3, 1.2) |
| Severe drought | 3.0*** (1.4, 4.7) | 2.9*** (1.3, 4.6) | 0.5 (-0.9, 1.9) | 0.3 (-1.0, 1.7) | 1.0** 0.2, 1.7) | 0.9* (0.2, 1.6) | 1.3** (0.5, 2.2) | 1.3** (0.4, 2.2) |
| Mozambique Removed | | | | | | | | |
| No drought | REF | REF | REF | REF | REF | REF | REF | REF |
| Moderate/ mild drought | 0.4 (-1.0, 1.4) | -0.1 (-1.2, 1.1) | 0.8 (-0.1, 1.7) | 0.7 (-0.2, 1.7) | 0.7** (0.2, 1.2) | 0.6** (0.2, 1.1) | 0.8** (0.3, 1.3) | 0.8** (0.3, 1.2) |
| Severe drought | 3.4*** (1.7, 5.1) | 3.2*** (1.6, 4.9) | 0.3 (-1.1, 1.7) | 0.1 (-1.3, 1.4) | 0.9* (0.1, 1.7) | 0.8* (0.1, 1.3) | 1.5** (0.6, 2.4) | 1.4** (0.5, 2.3) |
| Malawi Removed | | | | | | | | |
| No drought | REF | REF | REF | REF | REF | REF | REF | REF |
| Moderate/ mild drought | 0.3 (-1.0, 1.5) | -0.1 (-1.3, 1.1) | 0.1 (-0.8, 1.1) | 0.1 (-0.9, 1.1) | 0.7** (0.3, 1.2) | 0.7** (0.2, 1.2) | 0.7** (0.3, 1.2) | 0.7** (0.2, 1.2) |
| Severe drought | 3.3*** (1.6, 5.0) | 3.2*** (1.5, 4.9) | 0.5 (-0.9, 1.9) | 0.3 (-1.1, 1.8) | 0.9* (0.2, 1.7) | 0.9* (0.1, 1.6) | 1.4** (0.5, 2.4) | 1.4** (0.5, 2.3) |
| Namibia Removed | | | | | | | | |
| No drought | REF | REF | REF | REF | REF | REF | REF | REF |
| Moderate/ mild drought | 0.4 (-0.8, 1.6) | 0.1 (-1.0, 1.3) | 0.4 (-0.5, 1.3) | 0.4 (-0.5, 1.2) | 0.7** (0.3, 1.2) | 0.7** (0.2, 1.1) | 0.7** (0.3, 1.2) | 0.7** (0.2, 1.1) |
| Severe drought | 3.4*** (1.7, 5.1) | 3.4*** (1.7, 5.0) | 0.6 (-0.8, 1.9) | 0.4 (-1.0, 1.8) | 1.0** (0.2, 1.7) | 0.9* (0.2, 1.6) | 1.4** (0.5, 2.2) | 1.3** (0.4, 2.1) |
| Rwanda Removed | | | | | | | | |
| No drought | REF | REF | REF | REF | REF | REF | REF | REF |
| Moderate/ mild drought | 0.4 (-0.8, 1.6) | 0.1 (-1.1, 1.3) | 0.5 (-0.5, 1.4) | 0.4 (-0.5, 1.4) | 0.8** (0.3, 1.2) | 0.7** (0.2, 1.2) | 0.7** (0.3, 1.2) | 0.7** (0.2, 1.2) |
| Severe drought | 3.4*** (1.7, 5.1) | 3.5*** (1.8, 5.1) | 0.8 (-0.6, 2.2) | 0.6 (-0.7, 2.0) | 1.0* (0.2, 1.7) | 0.9* (0.2, 1.6) | 1.3** (0.5, 2.2) | 1.3** (0.4, 2.2) |
| Sierra Leone Removed | | | | | | | | |
| No drought | REF | REF | REF | REF | REF | REF | REF | REF |
| Moderate/ mild drought | 0.5 (-0.7, 1.7) | 0.2 (-1.0, 1.4) | 0.6 (-0.8, 2.0) | 0.3 (-0.5, 1.2) | 0.8** (0.3, 1.2) | 0.7** (0.2, 1.2) | 0.8** (0.3, 1.2) | 0.7** (0.3, 1.2) |
| Severe drought | 3.1*** (1.4, 4.8) | 3.1*** (1.4, 4.7) | 0.6 (-0.8, 1.9) | 0.4 (-1.0, 1.7) | 0.8* (0.2, 1.6) | 0.8* (0.2, 1.5) | 1.2** (0.4, 2.1) | 1.2** (0.3, 2.0) |
| Tanzania Removed | | | | | | | | |
| No drought | REF | REF | REF | REF | REF | REF | REF | REF |
| Moderate/ mild drought | 0.4 (-0.8, 1.6) | 0.2 (-1.0, 1.4) | 0.4 (-0.5, 1.3) | 0.4 (-0.4, 1.3) | 0.7** (0.2, 1.2) | 0.7** (0.2, 1.2) | 0.6* (0.1, 1.0) | 0.6* (0.1, 1.0) |
| Severe drought | 3.1*** (1.4, 4.7) | 3.0*** (1.4, 4.7) | 0.8 (-0.6, 2.1) | 0.6 (-0.8, 2.0) | 0.9* (0.2, 1.7) | 0.9* (0.1, 1.6) | 1.2** (0.4, 2.1) | 1.2** (0.3, 2.0) |
| Togo Removed | | | | | | | | |
| No drought | REF | REF | REF | REF | REF | REF | REF | REF |
| Moderate/ mild drought | 0.4 (-0.8, 1.5) | 0.1 (-1.0, 1.3) | 0.4 (-0.5, 1.3) | 0.3 (-0.5, 1.2) | 0.8** (0.3, 1.3) | 0.7** (0.2, 1.2) | 0.7** (0.2, 1.2) | 0.7** (0.2, 1.1) |
| Severe drought | 2.8** (1.1, 4.5) | 2.8** (1.1, 4.5) | 0.9 (-0.5, 2.3) | 0.8 (-0.7, 2.2) | 0.6 (-0.1, 1.4) | 0.6 (-0.2, 1.3) | 1.5** (0.6, 2.4) | 1.4** (0.5, 2.4) |
| Uganda Removed | | | | | | | | |
| No drought | REF | REF | REF | REF | REF | REF | REF | REF |
| Moderate/ mild drought | 0.5 (-0.7, 1.7) | 0.2 (-1.0, 1.3) | 0.3 (-0.6, 1.2) | 0.2 (-0.7, 1.1) | 0.8** (0.3, 1.2) | 0.7** (0.3, 1.2) | 0.8*** (0.4, 1.3) | 0.8*** (0.4, 1.3) |
| Severe drought | 3.2*** (1.5, 4.8) | 3.1*** (1.5, 4.7) | 0.5 (-0.8, 1.8) | 0.3 (-1.0, 1.7) | 0.9* (0.2, 1.6) | 0.8* (0.2, 1.5) | 1.3** (0.5, 2.1) | 1.3** (0.5, 2.1) |
| Zambia Removed | | | | | | | | |
| No drought | REF | REF | REF | REF | REF | REF | REF | REF |
| Moderate/ mild drought | 0.5 (-0.7, 1.8) | 0.2 (-1.1, 1.4) | 0.6 (-0.4, 1.6) | 0.6 (-0.4, 1.6) | 0.8** (0.3, 1.2) | 0.7** (0.2, 1.2) | 0.9*** (0.4, 1.4) | 0.9*** (0.4, 1.4) |
| Severe drought | 3.1*** (1.5, 4.8) | 3.1*** (1.4, 4.7) | 0.7 (-0.7, 2.1) | 0.5 (-0.9, 1.9) | 0.9* (0.2, 1.6) | 0.8* (0.1, 1.5) | 1.3** (0.5, 2.1) | 1.3** (0.5, 2.1) |
| Zimbabwe Removed | | | | | | | | |
| No drought | REF | REF | REF | REF | REF | REF | REF | REF |
| Moderate/ mild drought | 0.1 (-1.1, 1.4) | -0.2 (-1.4, 1.0) | 0.4 (-0.6, 1.3) | 0.3 (-0.7, 1.3) | 0.7** (0.3, 1.2) | 0.7** (0.2, 1.2) | 0.7*** (0.3, 1.2) | 0.7** (0.2, 1.2) |
| Severe drought | 2.9** (1.2, 4.5) | 2.8** (1.2, 4.4) | 0.5 (-0.9, 1.9) | 0.4 (-1.1, 1.7) | 0.9* (0.1, 1.6) | 0.8* (0.1, 1.5) | 1.2** (0.4, 2.1) | 1.2** (0.4, 2.0) |
| Coefficients are presented as marginal risk difference estimates in percentage points from logistic regression models with 95% confidence intervals in parentheses. The unadjusted model includes country-level fixed effects. The adjusted model includes age category, literacy, marital status, number of births, household size, rural, husband/partner’s age, and husband/partner’s education. Standard errors are clustered at the EA level.  Asterisks denote level of significance **p<0.01 **p<0.05 | | | | | | | | |
