# Supplementary material for: Drought and intimate partner violence towards women in 19 countries in sub-Saharan Africa during 2011-2018: A population-based study
Source: PLoS Med. 2020 Mar 19;17(3):e1003064. doi: 10.1371/journal.pmed.1003064 (PMC7081984; doi:10.1371/journal.pmed.1003064)
Supplement: S7 Table — (DOCX) [file pmed.1003064.s008.docx]

| **S7 Table. Associations between severe drought & moderate/mild drought and IPV among women aged 15-49 (n = 83,990) specified with multilevel logistic regression.** | | | | | | | | |
| --- | --- | --- | --- | --- | --- | --- | --- | --- |
|  | *Outcome* | | | | | | | |
|  | At least 1 control issue reported | | Emotional violence in previous 12 months | | Physical violence in previous 12 months | | Sexual violence in previous 12 months | |
| *Exposure* | Unadjusted | Adjusted | Unadjusted | Adjusted | Unadjusted | Adjusted | Unadjusted | Adjusted |
| No Drought | REF | REF | REF | REF | REF | REF | REF | REF |
| Moderate/ Mild Drought | 0.2 (-0.9, 1.4) | 0.0 (-1.2, 1.1) | 0.8 (-0.5, 2.0) | 0.6 (-0.6, 2.0) | 0.8** (0.2, 1.3) | 0.7** (0.2, 1.2) | 0.6** (0.2, 1.0) | 0.7** (0.3, 1.2) |
| Severe Drought | 2.7*** (1.1, 4.3) | 2.6*** (1.0, 4.2) | 0.6 (-0.2, 1.5) | 0.7 (-0.6, 2.0) | 0.8* (0.1, 1.6) | 0.7* (0.0, 1.5) | 1.1** (0.4, 1.7) | 1.0** (0.3, 1.7) |
| Coefficients are presented as marginal risk difference estimates in percentage points from multilevel logistic regression models (random intercepts at the country and enumeration area level) with 95% confidence intervals in parentheses. The unadjusted model includes country-level fixed effects. The adjusted model includes age category, literacy, marital status, number of births, household size, rural, husband/partner’s age, and husband/partner’s education. Standard errors are clustered at the EA level.  Asterisks denote level of significance ***p<0.001 **p<0.01 *p<0.05 | | | | | | | | |
