# Supplementary material for: Drought and intimate partner violence towards women in 19 countries in sub-Saharan Africa during 2011-2018: A population-based study
Source: PLoS Med. 2020 Mar 19;17(3):e1003064. doi: 10.1371/journal.pmed.1003064 (PMC7081984; doi:10.1371/journal.pmed.1003064)
Supplement: Attachment — Submitted filename: Drought_IPV_Response_vf.pdf [file pmed.1003064.s009.pdf]

Requests from the editors:

Please adapt your title so that the portion after the colon makes the study design (e.g. "a cross-sectional study") clear.

We have modified the title to *Drought and intimate partner violence: a population-based study from 19 countries in sub-Saharan Africa*

We ask you to note in the "methods and findings" subsection of your abstract the countries which do not fit with the pattern of "drought associated with IPV".

The following is now in the abstract: "In analyses stratified by country, we found three settings where drought was protective of at least one measure of IPV: Namibia, Tanzania, and Uganda."

Please add summary demographic details about study participants to your abstract.

The following is now in the abstract: "Of the 83,990 women included in the analytic sample, 10.7% (9,019) experienced severe drought and 23.4% (19,639) experienced moderate/mild drought in the year prior to the survey, with substantial heterogeneity across countries. The majority of women lived in rural areas (66.3%) and were married (73.3%), while less than half (42.6%) were literate."

Please add a new final sentence to the "methods and findings" subsection of your abstract, in which you summarize the study's main limitations.

The following is now in the abstract: "This study is limited by its lack of measured hypothesized mediating variables linking drought and IPV, prohibiting a formal mediation analysis."

Early in the methods section of your main text, please state whether or not the study had a protocol or prespecified analysis plan, and if so attach the document(s) as a supplementary file (referred to in the methods section). Please highlight analyses that were not prespecified.

All variables (covariates, outcomes, and exposure) were defined *a priori*. This is now explicitly stated in the text. In addition, although these statistical analyses were prespecified (and were reported to the Demographic and Health Surveys for data access), we did not document a formal analysis plan or pre-register this analysis.

To your methods section, please add a brief statement on ethics approval, which might state that specific approval was not required for the present study, for example.

We have added the following to the Methods:
"*Ethical Approval*

Demographic and Health Surveys obtain informed and voluntary consent from survey participants and permission to use DHS data was obtained from the DHS program. Specific approval for this de-identified secondary data analysis was not required."

Please add an additional sentence or two to the data statement so as to assist readers in locating and accessing the study data.
In your abstract and elsewhere, please include p values alongside CI where available.
After the abstract, we will need to ask you to add a new and accessible "author summary" section in non-identical prose. You may find it helpful to consult one or two recent research papers published in PLOS Medicine to get a sense of the preferred style.
At line 48, please add "In this study, we found that ..." or similar.
In the paragraph at lines 85-89, please use a consistent tense (i.e., "we used ... we evaluated").
Throughout your text, please format reference call-outs as follows: "... human activity [4,5].".
Please avoid "-0.0", as at line 234, for example.
Please ensure that all reference citations have full access information (e.g., reference 9).
References 29, 32 and 41 may need some additional information.
Please add a completed checklist for the most appropriate reporting guideline, which may be STROBE or RECORD, referred to in your methods section. In the checklist, individual items should be referred to by section (e.g., "Methods") and paragraph number rather than by page or line numbers, as the latter generally change in the event of publication.

We have fulfilled all of the edits requested above.

Comments from the reviewers:

*** Reviewer #1:

This is a very impressive and important study detailing the social and psychological impact of droughts in Sub-Saharan Africa. The authors have performed a very commendable job of using nationally-representative data from 19 countries and demonstrate that exposure to prolonged periods of drought is associated with different forms of interpersonal violence. It points to the need to address the emotional impacts of a very specific climate-related event which threaten the lives of women in particular and families in general as they struggle to cope with the economic consequences of such events.

There are a few suggestions offered that would improve the quality and impact of the manuscript, however.

First, the authors should probably document the number of people whose lives have been affected by drought in Sub-Saharan Africa, both today and during the study period. Such information is readily available from sources such as the UN and the WHO.

We have added numbers of individuals exposed to drought in sub-Saharan Africa to the Introduction, paragraph 1: "In 2014-2016, Southern Africa experienced two years of an El Niño event-induced drought, leading to national emergency declarations in a number of countries and exposing 38 million people across the region [7]. In 2019, the number of

individuals exposed to severe drought in one year in sub-Saharan Africa swelled to 45 million [8].”

Second, IPV was assessed using four binary indicators, but this raises the question as to whether someone could experience more than one form of IPV. It would be helpful if the authors examined whether there was a dose-response relationship between extent of drought and number of IPV indicators or explain why such a comparison is not meaningful or possible with the data.

Thank you for suggesting this. Below we present an analysis using ordered logistic regression with a count outcome of the number of IPV outcomes endorsed (range 0-4). We include this now as S5 Table, but will include it in the manuscript at the request of editors or the Reviewer.

Exposure	Unadjusted	Adjusted
	Odds ratio (95% CI)	Odds ratio (95% CI)
No Drought	REF	REF
Moderate/ Mild Drought	1.04 (1.00, 1.09)	1.03 (0.99, 1.08)
Severe Drought	1.15*** (1.08, 1.24)	1.14*** (1.07, 1.23)
Coefficients are presented as odds ratios from order logistic regression models with 95% confidence intervals in parentheses. The unadjusted model includes country-level fixed effects. The adjusted model includes age category, literacy, marital status, number of births, household size, rural, husband/partner’s age, and husband/partner’s education. Standard errors are clustered at the EA level. Asterisks denote level of significance ***p<0.001 **p<0.01 *p<0.05		

Third, while Figure 2 illustrates differences in drought level by country, there are also important differences in drought level within each country during the study period. Women in some of these countries may have been exposed to longer periods of drought than others but the results do not appear to reflect this. If the ranking of quantity of precipitation does reflect these differences, then the authors should explain how it does so.

This method used to quantify drought in this manuscript, employed elsewhere in the drought and health outcomes literature, ranks the most recent 12 months of precipitation relative to the previous 29 years. It does not, however, take into consideration cumulative exposure to drought (e.g., repeated droughts over the years prior to the survey). Although evaluating these associations with repeated exposures is warranted, it is not possible with the precipitation data available. It is recommended that at least 30 years of historical rainfall information is used in order to define precipitation shocks. CHIRPS precipitation data starts in 1981; therefore, the earliest year that drought can be estimated using our definition is 2011. For surveys that took place in 2011 we can only estimate drought in the year before the survey, for those that took place in 2012 we can estimate only the 2 years leading up to the survey, etc.

Fourth, the proposed conceptual framework presented in the discussion section on p. 19 should be eliminated as the study provided no results supporting it. A discussion of potential causal

mechanisms is appropriate, but presentation of a conceptual framework such as the one proposed in the manuscript is premature.

We have removed Figure 4 and have kept the discussion of potential pathways in the text body.

Fifth, while the inclusion of 19 countries may enhance the external validity of the study findings, it also increases the likelihood of ignoring important cultural differences within those 19 countries. This should be addressed as a potential limitation as cultural differences may result in different patterns of reporting of the different forms of IPV. While drought may reflect a reprieve in Uganda and Tanzania, it does not appear to be the case in Namibia.

Thank you for pointing this out. We have added in a note on the potential for misclassification differentially between countries in the Limitations, paragraph 2: "Third, the IPV outcomes may be misclassified because women tend to underreport experiences of IPV, which could affect the magnitude of associations and due to cultural differences in reporting IPV across regions and countries. However, we do not believe that reporting bias would depend on exposure status within countries and therefore any bias would be toward the null on average, suggesting that our results underestimate the magnitude of true associations."

*** Reviewer #2:

This is a well-written study investigating ecological associations at a country level between drought and IPV in sub-Saharan Africa. The introduction presents a clear justification for the study. The methods are reported in appropriate detail and there are good measures of drought rates and sensitive measures of IPV across 4 outcomes (being controlled, victim of violence, emotional abuse, and sexual violence). These IPV-related outcomes are based on self-report measures and the authors excluded 31% of participants who were asked questions in the domestic violence module of a questionnaire as they were not married or living with a partner.

Some major areas that need clarification:

1. Why did the authors exclude these 31% of participants? Is it the case that only those who are married or living with a male partner can experience IPV? I would assume that intimate relationships (and therefore IPV) can exist outside of these inclusion criteria, and thus the IPV estimates reported in the paper may be biased one way or the other due to this exclusion (and I would suspect that they are biased upwards).

The DHS surveys ask the IPV questions only to women who are married or cohabitating. These questions are not asked to women who are not married or cohabitating, and hence they could not be included for this study question. We agree with the reviewer that the lack of data on non-married, non-cohabitating women is a limitation, which we now expand upon in the Limitations section, paragraph 2: "Sixth, the IPV questions were only asked of married and cohabitating women, and hence the results are not generalizable to women with a different relationship status. Since non-married, non-cohabitating women

are also at risk for violence, future studies should assess these associations among all women."

2. The covariates were categorized a priori. Was this the same for other variables (e.g. drought, IPV-related outcomes)? Was there a statistical plan?

Yes, all variables were categorized a priori, including the exposure and outcome variables. We have now clarified this in the text, under Measures: "The calculation and classifications of drought were specified *a priori*," and, "We considered four binary outcomes selected prior to analysis, each representing a different dimension of risk of and/or experienced IPV…" We followed a pre-specified statistical analysis plan.

3. The findings appear to show considerable between-country heterogeneity, which would question their decision to pool findings and present overall marginal risk differences. Can they estimate the degree of heterogeneity? Looking at control-related outcome, 11 countries had no clear relationship with drought, 2 were negatively associated, and 6 were positively associated. This is too much statistical heterogeneity in my view for a pooled estimate.

We have now added estimates for the degree of heterogeneity by specifying drought-country interactions and reporting the p-value of the joint interaction term, Results, paragraph 4: "The results were significantly heterogeneous between country for reporting a controlling partner (P for joint interaction term < 0.0001), emotional violence (P for joint interaction term = 0.0085), and physical violence (P for joint interaction term = 0.022), but not for reported sexual violence (P for joint interaction term = 0.40)."
We agree that there is a great deal of heterogeneity in these estimates. In order to account for this heterogeneity, we also specified random effects (rather than fixed effects) models with random intercepts at the country level. The results were consistent with the fixed effects models. These results are now discussed in Results, paragraph 4, and shown in S7 Table. We believe that presenting both country-level and pooled estimates allows the reader to observe regional differences in addition to the weighted average (pooled association) and to draw their own conclusions.

4. There is also heterogeneity across the 4 IPV-related outcomes - in that emotional outcomes are not associated with drought, unlike pooled estimates for sexual violence or interpersonal violence.

Thank you for noting this finding; we agree it requires more attention in the text. We have now added the following discussion of this finding, Discussion, paragraph 6: "We did not find evidence for an association between drought and emotional violence in the pooled sample, contrary to our hypothesis. This may be because emotional violence is less clearly defined than physical and sexual violence and is therefore more prone to measurement error, leading to an attenuation of the association."

5. The conclusion starts referring to 'increased risk' whereas the results discussed marginal risk differences. Consistency in the presentation of the findings is required.

Thank you for pointing this out. We now use the term "higher" instead of "increase" in order to remove causal language. These changes can be found in the Discussion, paragraph 1.

Overall, with the substantial between-country and across-outcome heterogeneities, I think that the findings do not warrant the relatively firm conclusions drawn. Other measures of IPV would be helpful to triangulate the findings, which as they stand are hypothesis-generating.

We agree that the language used in the manuscript needs to be toned down to account for the heterogeneity in the findings. We have made edits to the Abstract and Discussion underscoring the heterogeneity.

*** Reviewer #3:

This review considers the use of statistics in the paper by Epstein and colleagues, which investigates the association between periods of drought and reports of intimate partner violence in sub-Saharan Africa.

Overall, I think the statistical elements of the paper are very good. The data sources are well described, and the use of logistic regression to assess the association of interest is perfectly reasonable. Presenting the results on an absolute scale, rather than as odds ratios, is an interesting approach, and perfectly reasonable. Use of interactions to assess variations in associations between subgroups is good.

The comments that I have are generally quite minor.

Line 140 mentions the minimum household size as being 1, when I think it should be 2. I saw the same thing in Appendix S4.
Line 199 uses the word "relationship", when "association" is slightly preferable, to avoid any implication of causality.

Thank you for pointing us to these errors. They have been corrected.

Figure 3 shows country-specific associations. Are these derived from separate models, with different covariate effects in each model, or from models with country-by-drought interactions? Are these associations significantly different? I suspect they are, but a p-value would help.

We have added in a clarification that we considered countries as effect measure modifiers and therefore estimated their country-level associations using an interaction term. We report the significance of the joint interaction terms in the Results, paragraph 4: "The results were significantly heterogeneous between country for reporting a controlling partner (P for joint interaction term < 0.0001), emotional violence (P for joint interaction term $= 0.0085$), and physical violence (P for joint interaction term $= 0.022$), but not for reported sexual violence (P for joint interaction term $= 0.40$)."

Line 291 raised an interesting point. Does this mean that it was not possible from the survey data

used, to identify women who had experienced IPV in the previous 12 months, but had left the household by the time of the survey? Even if this were possible, it would probably also be necessary to identify women who had left a partner in the past 12 months without experiencing IPV, and I can imagine this would be more difficult.

We agree that this is a limitation of the data source and presents a challenge with studying migration using cross-sectional data.
