# Supplementary material for: Drought and intimate partner violence towards women in 19 countries in sub-Saharan Africa during 2011-2018: A population-based study
Source: PLoS Med. 2020 Mar 19;17(3):e1003064. doi: 10.1371/journal.pmed.1003064 (PMC7081984; doi:10.1371/journal.pmed.1003064)
Supplement: Attachment — Submitted filename: Drought_IPV_ResponseLetter_2.docx [file pmed.1003064.s010.docx]

Requests from Editors:

Please add to your conflict of interest statement that SDW is a member of PLOS Medicine's editorial board.

**This has been done.**

We ask you to restructure the title to better match journal style, and suggest "Drought and intimate partner violence in 19 countries in sub-Saharan Africa during 2011-2018: a population-based study".

At line 65, we suggest amending the text to "... intimate partner violence towards women." or similar. We suggest making similar amendments elsewhere in your ms, e.g. at line 80 and in the title.

**The title is now: “Drought and intimate partner violence towards women in 19 countries in sub-Saharan Africa during 2011-2018: a population-based study”**

**We have also clarified IPV “towards women” throughout the manuscript.**

Can you quote a mean age for study participants around line 40?
**The abstract now includes the sentence: “The mean age of respondents was 30.8 (standard deviation 8.2).”**

To the sentence at the end of the "methods and findings" subsection of your abstract summarizing study limitations, we ask you to add a few words to mention 1-2 further limitations, such as the possibility of unmeasured confounding affecting the results.

**The final sentence of “Methods and Findings” subsection of the abstract now reads: “Additional limitations include the potential for bias due to residual confounding and potential non-differential misclassification of the outcome measures leading to an attenuation of observed associations.”**

Around line 162 of your main text, please state explicitly that your study did not have a written prespecified analysis plan or protocol.

**This is now reflected in the text.**

Around line 393, we suggest mentioning possible unmeasured confounding in the list of potential study limitations.
**Find the following on page 21: “Eighth, given the observational nature of the data, residual confounding may bias the observed associations. However, by using EA-level deviations from long-term precipitation as the definition of drought, the possible variation related to socio-demographic factors associated with historically dryer or wetter places that may also impact IPV should be removed, and therefore we do not expect confounding to be substantial.”**

Please add an author or group name to reference 8.
Please add additional access information to references 1 and 37 as needed.
Noting references 29 and 30, for example, please ensure that journal names are abbreviated as appropriate in your reference list.
**The errors with the references have been rectified. However, please note that Reference 1 is an online journal; there are no issue or page numbers.**

Comments from Reviewers:

*** Reviewer #1:

The revised manuscript appears somewhat responsive to the reviews.  The authors appear to have misunderstood recommendation 2 provided by Reviewer 1. Given that there are four different indicators of IPV, the question is whether greater exposure to drought results in more than one indicator (e.g., control plus physical violence plus emotional violence). This was not addressed by the authors.

**We implemented this suggestion using ordered logistic regression; the results can be found in S5 Table. The outcome in this model is a count of the number of IPV outcomes the respondent endorsed. The exposure is the categorical classification of drought (no drought, moderate/mild drought, and severe drought). The results in the table below suggest that women living in severe drought had 1.14-times the odds of endorsing an IPV outcome compared to those not living in drought.**

| **S5 Table. Associations between drought and number of IPV outcomes endorsed among all women aged 15-49 in pooled analysis.** | | |
| --- | --- | --- |
| *Exposure* | Unadjusted | Adjusted |
|  | Odds ratio (95% CI) | Odds ratio (95% CI) |
| No Drought | REF | REF |
| Moderate/ Mild Drought | 1.04 (1.00, 1.09) | 1.03 (0.99, 1.08) |
| Severe Drought | 1.15*** (1.08, 1.24) | 1.14*** (1.07, 1.23) |
| Coefficients are presented as odds ratios from ordered logistic regression models with 95% confidence intervals in parentheses. The unadjusted model includes country-level fixed effects. The adjusted model includes age category, literacy, marital status, number of births, household size, rural, husband/partner’s age, and husband/partner’s education. Standard errors are clustered at the EA level.  Asterisks denote level of significance ***p<0.001 **p<0.01 *p<0.05 | | |

Second, given the inability to assess duration of exposure to drought with the data available, the authors should note this as a limitation to the study because it weakens the finding of an association between drought and IPV.
**Thank you for suggesting this. We have added the following to the limitations on page 21: “Seventh, we were unable to assess the impact of repeated exposure to drought given the availability of CHIRPS rainfall data.”**

*** Reviewer #2:

The authors have responded to most of the queries comprehensively. However, there is one issue that needs further consideration - the between-country heterogeneity has not been sufficiently addressed in the revision in my view. There is no mention of this major limitation in the discussion.

Further, the large heterogeneity would argue against having a pooled estimate in Fig 3 - and ranges can be reported instead.

**There is clearly heterogeneity across countries in terms of the association between drought and the outcomes. However, we still believe there is value to presenting both country-level and pooled associations, allowing the reader to understand both and come to their own judgment.**

**Presenting both pooled and country-level estimates in the presence of effect estimate heterogeneity is common in the literature with Demographic and Health Survey data. See a brief sample of recent citations below.**

**Amouzou A, Jiwani SS, da Silva ICM Countdown 2030 Coverage Technical Working Group, et al. Closing the inequality gaps in reproductive, maternal, newborn and child health coverage: slow and fast progressors. BMJ Global Health 2020;5:e002230.**

**Yaya S, Uthman OA, Kunnuji M, et al. Does economic growth reduce childhood stunting? A multicountry analysis of 89 Demographic and Health Surveys in sub-Saharan Africa. BMJ Global Health 2020;5:e002042.**

**Efevbera, Y., Bhabha, J., Farmer, P. *et al.* Girl child marriage, socioeconomic status, and undernutrition: evidence from 35 countries in Sub-Saharan Africa. BMC Med 2019;17, 55.**

The authors have presented p values for the joint interaction term to estimate the degree of heterogeneity - but it is unusual and I would have expected to see I squared (which are easier to interpret and more widely used). They have argued that they have used a random effects model to account for this - but this is not considered appropriate for observational data if the heterogeneity is substantial.

**Thank you for noting this. Indeed, I-squared is a commonly used metric reported in meta-analyses. However, we have opted to report the P value for joint interaction terms as suggested by Reviewer #3 (the statistical reviewer in the previous round of peer review). This is because, although similar in theory, we are not performing a true meta-analysis of different effect estimates from various studies, but rather pooling data across countries from the same source.**

**For our original analysis, we performed fixed effects logistic regression. This method takes into account only within-country variation. Reviewer #2 noted this was inappropriate given the degree of between-country heterogeneity; we then performed random effects logistic regression which takes into consideration both within and between-country variation. Please note that the results did not change qualitatively when random effects were implemented.**
